# Treatment experiences, preferences, and expectations for cognitive impairments in long COVID among Chinese young and older adults: a constructivist grounded theory study

**DOI:** 10.1186/s12916-025-04457-5

**Published:** 2025-10-23

**Authors:** Dan Shan, Carol Holland, Trevor J. Crawford

**Affiliations:** 1 Centre for Ageing Research, Division of Health Research, Faculty of Health and Medicine, Lancaster University, Health Innovation Campus, Sir John Fisher Drive, Bailrigg, Lancaster, LA1 4YT UK; 2 Centre for Ageing Research, Department of Psychology, Faculty of Science and Technology, Lancaster University, Lancaster, UK

**Keywords:** COVID, Long COVID, Brain fog, Cognitive impairment, Patient preference, Qualitative study, Patient-centred care, Grounded theory

## Abstract

**Background:**

Cognitive impairments associated with long COVID disrupt daily functioning and psychological well-being. While increasing research has examined prevalence and mechanisms, little is known about patients’ treatment experiences, preferences, and expectations. In the absence of validated effective treatments, integrating these perspectives is essential for guiding research priorities and clinical trial design. In China, where long COVID is an emerging public health concern, awareness of cognitive impairments remains limited and access to specialised care is inadequate. Considering potentially substantial differences in baseline health and treatment expectations between young and older adults, this study aimed to explore and compare their perspectives using a qualitative approach.

**Methods:**

We adopted constructivist grounded theory to capture participants’ lived experiences and develop a theory grounded in their narratives. Semi-structured online interviews were conducted with 23 individuals recruited via Chinese social media long COVID mutual aid groups, including 10 young adults (18–39 years) and 13 older adults (≥ 60 years). Theoretical sampling guided recruitment and iterative analysis through initial, focused, and theoretical coding, leading to the development of a framework explaining treatment preferences and expectations.

**Results:**

All participants reported cognitive impairments based on self-perception, with no formal medical diagnoses. We constructed a theoretical framework of “Individualised and Dynamic Adaptation to Cognitive Challenges”. Preferences and expectations could be shaped by age, symptom severity, prior management experiences, lifestyle, doctor–patient interactions, and health literacy. Young adults showed a strong preference for non-pharmacological strategies, including self-directed approaches and emotional support to address stigma. Older adults emphasised a balanced use of pharmacological and non-pharmacological interventions, supported by family and structured routines, while expressing holistic expectations that encompassed cognitive, physical, and emotional well-being. Across both groups, improved sleep and psychological health were consistently emphasised.

**Conclusions:**

Age-specific differences highlighted the heterogeneity of long COVID experiences and underscored the need for dynamic, patient-centred approaches. Tailored interventions that integrate patient perspectives may enhance care quality and outcomes. Holistic care, particularly for older adults who may face additional comorbidities and functional challenges, is essential. In China, increasing awareness among the public and healthcare providers, reducing stigma, and addressing inequalities in care access should be prioritised.

**Supplementary Information:**

The online version contains supplementary material available at 10.1186/s12916-025-04457-5.

## Background

Long-lasting symptoms following Coronavirus Disease 2019 (COVID-19) infection, commonly referred to as “long COVID” [1], have emerged as one of the most detrimental consequences of the virus. Recent global-to-local analyses of COVID-19 burden and long COVID vulnerability have highlighted substantial and unequal impacts across regions and populations, underscoring the urgency of addressing cognitive sequelae in diverse contexts [2, 3]. Long COVID is characterised by a range of symptoms that typically arise within 3 months of the initial Severe Acute Respiratory Syndrome Coronavirus 2 (SARS-CoV-2) infection and persist for at least 2 months beyond the acute phase, without an identifiable alternative explanation [4]. Although symptoms are diverse, many individuals experience cognitive impairments such as memory loss, attention deficits, and executive dysfunction, which significantly challenge recovery and rehabilitation across age groups [5–7].

An online survey study in an international cohort across all age groups revealed that cognitive difficulties and memory problems were the most common and persisting neurological symptoms in people with long COVID, with 58.4% of them still experiencing cognitive dysfunction 6 months after symptom onset [8]. Ageing is the biggest risk factor for cognitive impairment and dementia [9] and may render older adults more susceptible to post-COVID cognitive effects than younger individuals. A recent meta-analysis showed that older adult COVID-19 survivors (≥ 60 years old) were 84% more likely to develop new-onset dementia 12 months after COVID-19 infection compared to non-COVID-19 individuals with otherwise unspecified health status [10, 11]. Given the irreversible nature and severe impact of dementia [12], these findings underscore the urgent need for interventions to address lingering cognitive deficits in individuals with long COVID-associated impairments, particularly among older adults [13], who are inherently at higher risk for dementia, to prevent progression to this condition. In this context, prior evidence suggested that although older adults with mild cognitive impairment (MCI) experienced cognitive deficits, they retained a degree of brain plasticity, which allowed for potential functional improvement through targeted interventions [14]. Since COVID-19 could accelerate cognitive decline and increase the risk of progression from MCI to dementia, leveraging this preserved plasticity was considered crucial for mitigating COVID-related cognitive deterioration. However, it should be noted that long COVID-associated cognitive impairments may also substantially impact young adults (e.g. loss of employment or decline in study performance) [15, 16], indicating that this population should also be a concern for the public.

Although spontaneous improvement in long COVID cognitive symptoms is possible [17], targeted interventions may expedite recovery. Existing therapeutic approaches include pharmacological treatments (e.g. N-acetyl-cysteine and guanfacine) [18]; cognitive behavioural therapy (CBT) [19]; physical activity rehabilitation [20]; lifestyle modifications such as dietary adjustments and controlled breathing exercises [21]; and other approaches [22]. However, the efficacy of these interventions remains uncertain, as prior studies have reported mixed results [23, 24]. These inconsistencies may result from factors such as varying sample sizes, heterogeneity in participant characteristics, differences in inclusion criteria for long COVID-associated cognitive impairments, reliance on subjective patient-reported outcomes versus objectively assessed measures, and variations in follow-up durations across trials [23, 24].

For example, while physical exercise has demonstrated cognitive benefits outside the context of COVID-19 [24, 25], the same does not always hold true for long COVID cases [24]. Many individuals with long COVID report post-exertional malaise (PEM)—an exacerbation of symptoms including fatigue, challenges in regulating body temperature, and cognitive impairments, following even light exercise [24, 26]. As a result, for patients experiencing PEM, alternative methods of stimulating cognition may be preferable, as exercise could worsen their symptoms [24]. Even when exercise therapy shows potential cognitive benefits, individuals with long COVID are likely to hesitate to engage in physical activity due to negative experiences with previous attempts. Therefore, from a patient-centred care perspective, the optimal management strategy for cognitive impairments related to long COVID should combine evidence-based interventions with a personalised approach. This involves integrating patients’ perspectives, addressing their individual experiences and preferences, and tailoring interventions to the heterogeneity of cognitive symptoms and associated risks, such as PEM.

Patient-led research initiatives emphasize the value of incorporating real-world experiences to address the complexities of long COVID. For instance, surveys conducted by the Patient-led research collaborative (PLRC) identified over 200 symptoms, including cognitive impairments such as brain fog, and underscored the need for targeted interventions informed by patient perspectives [27]. These efforts have also encouraged investigations into potential treatments for long COVID, such as low-dose naltrexone, which some individuals report as alleviating symptoms, possibly due to its anti-inflammatory and pain-relieving effects [27]. Such collaborations highlight the importance of patient involvement in shaping effective, personalised strategies for managing long COVID symptoms [28]. Qualitative research that explores patients’ lived experiences and treatment preferences is crucial for designing interventions that genuinely address their actual needs. This patient-centred approach ensures holistic care and aligns treatments with individual priorities, enhancing both clinical efficacy and patient satisfaction.

Health-related factors, particularly ageing, may influence the preferred approaches to cognitive care in long COVID. Variations in residual physiological capacity, baseline health status, disrupted activities of daily living, recovery expectations, and personal needs between younger and older adult patients suggest that treatment preferences may differ across age groups. Both younger and older age groups might, for certain reasons, avoid specific rehabilitation paths even when such methods show potential for alleviating persistent cognitive symptoms. For instance, younger adults, often possessing greater baseline endurance and cognitive resilience, might prefer non-pharmacological options such as CBT and aerobic exercise, particularly if they do not experience post-exertional malaise, and might be wary of long-term medication use due to potential side effects [29, 30]. In contrast, older adults, who usually have lower physiological reserves and more comorbidities, might prefer approaches that minimise physical and cognitive strain, such as low-intensity cognitive exercises or carefully monitored pharmacological options, prioritising safety and tolerability [31, 32]. These differences also underscore the need for patient-centred care that aligns therapeutic strategies with individual preferences and expectations [27]. Evidence indicates that active patient participation in decision-making enhances compliance, satisfaction, and treatment effectiveness [33, 34].

At present, no widely validated treatments exist for managing cognitive impairments associated with long COVID. To allocate limited research resources efficiently towards exploring potentially effective interventions, it is essential to ground these efforts in patient perspectives to avoid inefficiencies and suboptimal outcomes. The complexity and heterogeneity of long COVID-associated cognitive impairments necessitate an approach that allows for an in-depth exploration of individual experiences, while accounting for the interplay of diverse personal, social, and systemic influences. Moreover, pandemic-related factors such as extended lockdowns and prolonged social isolation may also have contributed to perceived cognitive decline and reduced well-being in both younger and older adults [35]. These contextual influences should be taken into account when interpreting long COVID-related cognitive impairments. Furthermore, research has shown that patient expectations could influence treatment outcomes in various conditions, including cognitive issues. When patients perceive a treatment as being aligned with their beliefs and expectations, and when their perspectives are heard and respected during the rehabilitation process, they are more likely to engage with it and experience better outcomes, particularly when there are low predictive errors between their expectations and actual experiences following treatment [36, 37]. Understanding and integrating patient expectations is therefore critical for optimizing long COVID cognitive rehabilitation.

Long COVID poses a significant health crisis in China. A large-scale survey of 74,075 Chinese participants found that approximately 10%−30% reported experiencing long COVID symptoms such as fatigue (30.53%), memory decline (27.93%), decreased exercise ability (18.29%), and brain fog (16.87%) [38, 39]. However, publicly available, well-evidenced platforms providing information on long COVID symptoms remain scarce, leaving the public with limited access to reliable information and low awareness of the conditions [40]. Additionally, the lack of specialised long COVID clinics in China further restricts access to appropriate care [40]. Hence, prioritizing research tailored to the needs of those with long COVID-associated cognitive impairments in China is essential to raise public awareness and lay the groundwork for identifying potentially effective interventions.

Thus, this study aimed to achieve several objectives. First, it sought to qualitatively identify the range of cognitive symptoms reported by Chinese individuals experiencing long COVID-related cognitive impairments, along with their past management experiences (whether through healthcare providers, self-management, or a combination of both). More importantly, the study aimed to explore treatment preferences and expectations, focusing on both their nature and the factors that shape them. Furthermore, it sought to compare these preferences and expectations between Chinese younger and older adults (given their likely differences in baseline health status), while also identifying other influential factors beyond age. To achieve these goals, the study aimed to employ a constructivist grounded theory methodology, which is well-suited for understanding participants’ perspectives and developing a theoretical framework based on their narratives. This approach emphasizes iterative data analysis, constant comparison, and theory generation, distinguishing it from thematic analysis, which primarily identifies and reports patterns in data without necessarily constructing a theoretical framework [41]. Ultimately, the study aimed to develop a theoretical framework explaining these processes, which could inform the design of patient-centred interventional trials focused on effective cognitive rehabilitation and recovery strategies.

## Methods

We used a qualitative research design to explore treatment experiences, preferences, and expectations regarding cognitive impairments among Chinese individuals with long COVID, focusing on both young and older adults. Semi-structured online interviews were conducted, and the data were analysed using a constructivist grounded theory approach, primarily to identify core themes and develop a theoretical framework that provides evidence for designing future patient-centred care intervention strategies. This study was approved by Lancaster University’s Faculty of Health and Medicine Research Ethics Committee (FHM-2025–4644-SA-2).

### Participant recruitment

We attempted to recruit participants from community-based samples, specifically through Long COVID mutual aid groups on Chinese social media platforms, including *WeChat* and *RedNote*. Due to the limited awareness of long COVID and its associated cognitive impairments among healthcare providers and few established diagnostic guidelines in China [40], few people experiencing long COVID-related cognitive impairments receive a formal diagnosis. Hence, we aimed to include both individuals who self-identified and those who informed us that they had been verified by hospitals or primary care physicians as having long COVID-related cognitive impairments (e.g. brain fog). Our recruitment efforts targeted 16–24 participants: 8–12 young adults (aged 18–39 years) and 8–12 older adults (aged 60 and above), with an aim for gender balance within each group. Only participants who met these criteria in line with our aims and were capable of participating in online interviews were included, whereas those unable to provide informed consent or actively engage in the interviews were excluded. Here, “capable of participating” referred to the ability to access the online interview platform either independently or with minimal assistance from family members to log into the interview, and “actively engage” referred to having no hearing or other difficulties that would have prevented continuation of the interview.

The determination of participant numbers across demographics (young vs. older adults) was guided by the principles of theoretical sampling in grounded theory. Rather than predetermining a fixed sample size, theoretical sampling facilitated an iterative process of participant selection driven by emerging themes and concepts, ensuring that diverse perspectives informed the development of the theory. Achieving theoretical saturation was a key goal, defined as the stage where no new themes or theoretical insights emerged. This dynamic sampling process evolved in response to ongoing analysis, addressing conceptual gaps and refining the theoretical constructs [42, 43]. As data were continuously monitored and analysed, recruitment was adjusted as needed to delve deeper into emerging themes and theories, ensuring the theoretical framework was thoroughly developed and remained robust.

### Semi-structured interviews

Given the complexity and diversity of experiences among individuals with long COVID, this study employed semi-structured interviews as the primary method for collecting detailed and personal insights. This approach ensured both consistency and flexibility: while predefined domains and guiding questions structured the interviews, the interviewer also used probing and follow-up questions to allow participants to elaborate freely on their experiences. The structured component of the interview guide covered five domains: (1) experiences with cognitive impairments (e.g. “Can you describe your experiences with any cognitive symptoms since having long COVID, such as brain fog?” and “Have you ever received a formal diagnosis of long-term COVID, or were your symptoms fully self-perceived?”), (2) treatment experiences (e.g. “What approaches have you tried to manage your symptoms, and what motivated these choices?”), (3) preferences and expectations (e.g. “What factors are most important to you when choosing how to manage your symptoms?”), (4) comparative perspectives across age (e.g. “Do you feel that age shapes the way you or others in your community handle these health challenges?”), and (5) future expectations (e.g. “What are your hopes or concerns regarding future strategies for managing symptoms like yours?”). The interview guide was iteratively refined based on early findings to address emerging themes and gaps in understanding [44].

Data collection was conducted exclusively through a secure online platform, *Tencent Meeting* (the Chinese equivalent of Zoom), to ensure safety and convenience. Each interview was conducted as a one-on-one session, aimed to last approximately 30–45 min, to allow for an in-depth exploration of participants’ experiences and perspectives. Upon completion, all interviews were transcribed and pseudo-anonymised to protect participants’ privacy, ensuring confidentiality during subsequent analyses.

The first author (D.S.), a junior researcher with formal training in qualitative methods (including constructivist grounded theory and semi-structured interviewing), conducted all interviews. Being a native Mandarin speaker fluent in English, D.S. was able to facilitate effective communication with participants and accurately translate the data for analysis and manuscript preparation. All interviews were conducted in Mandarin Chinese, the participants’ native language, to ensure they could fully and comfortably articulate their experiences. To minimise potential loss of meaning, the English transcripts were repeatedly checked against the original Mandarin transcripts and audio recordings. Where cultural or linguistic expressions lacked direct English equivalents, explanatory notes were added to preserve nuance. To further minimise potential bias, D.S. maintained reflexive notes throughout the data collection and translation process and engaged in regular discussions with the senior authors (C.H. and T.C.). The audio recordings were securely stored and deleted once they were no longer required for accuracy checks, further safeguarding participants’ privacy.

### Data analysis procedure

Grounded theory guided the data analysis, employing a systematic and iterative process to identify, refine, and integrate key concepts and their relationships. The analysis proceeded through three main stages: initial coding, focused coding, and theoretical coding, allowing for the development of a theoretical framework directly grounded in participants’ narratives [45]. The initial coding phase involved systematically breaking down textual data into discrete, meaningful segments and labelling them to capture initial concepts. In the focused coding stage, the most significant and insightful concepts were refined and synthesised, revealing patterns (e.g. themes, categories) while allowing for multiple interpretations. Finally, theoretical coding integrated these themes or categories into a cohesive theoretical framework, emphasizing core themes that explained the underlying patterns and dynamics in the data. NVivo software was used to support data management, coding, and retrieval, facilitating iterative review and refinement. It served solely as an organisational tool, while all coding decisions, category development, and theoretical integration were conducted by the research team in line with the constructivist grounded theory approach. Reflexive notes and regular team discussions ensured that interpretation and meaning-making remained researcher-driven. Theoretical saturation, defined as the point when no new theoretical insights or themes emerge from additional data collection, was used as a benchmark to determine the adequacy of data coverage [46].

In line with the constructivist grounded theory approach, we acknowledged that translation and interpretation were not neutral processes but part of the co-construction of meaning between participants and researchers. The first author (D.S.) maintained reflexive notes throughout data collection, transcription, translation, and analysis, documenting interpretative choices and reflecting on the dual positionality—as both an insider (shared language and cultural background) and an outsider (research training in the UK)—and how this might have shaped the analytic process. Reflexive discussions within the research team (C.H. and T.C.), both experienced qualitative researchers, were held regularly to critically examine coding decisions, emerging categories, and theoretical interpretations. These steps enhanced transparency, minimised potential bias, and ensured that the final framework reflected both participants’ voices and the team’s reflexive engagement.

### Patient and public involvement

Prior to data collection, the pre-set interview guide was reviewed by two individuals with lived experience of long COVID-associated cognitive impairments: one younger adult in their 30 s and one older adult in their 60 s, both Mandarin speakers residing in China. Their feedback focused on the clarity, comprehensibility, and relevance of the questions to everyday experiences. Minor wording adjustments were made accordingly to improve accessibility. The study findings will be disseminated through multiple channels. For healthcare professionals and academics, findings will be presented at national and international conferences related to ageing, public health, and post-COVID care. For the general public, accessible summaries will be shared via social media platforms commonly used in China, particularly targeting long COVID support groups and wider community audiences.

## Results

### Participant characteristics

Initially, 12 eligible young adults (6 men and 6 women) and 12 eligible older adults (6 men and 6 women) were successfully recruited and interviewed based on the inclusion criteria. Although the data collected from the 12 young adults initially appeared to achieve theoretical saturation (as no new theoretical insights or themes emerged), 2 participants later withdrew from the study. They expressed concerns that some perspectives shared during the interviews were sensitive (e.g. related to stigma surrounding long COVID in China), from their point of view, and felt it would be inappropriate to include their data in the study. Despite their withdrawal, theoretical saturation was still maintained. However, to ensure theoretical saturation among older adults, one additional participant was recruited and interviewed to address concerns that certain aspects of holistic care expectations may not have been fully captured in previous interviews. This ultimately confirmed saturation in the analysis. As a result, the whole analysis process included data from 10 young adults and 13 older adults. The average interview duration was 39.1 min for young adults (range, 31–55 min) and 37.5 min for older adults (range, 30–57 min), with an overall average of 38.2 min. The mean age was 31.2 years for young adults (range, 25–37 years, standard deviation = 4.0) and 70.8 years for older adults (range, 61–78 years, SD = 4.7). Although physician-confirmed cases were targeted during recruitment, none were identified, as all participants reported their cognitive impairments based on self-perception only. This outcome might reflect the current context in China, where limited awareness among healthcare providers and the absence of established diagnostic guidelines have meant that formal diagnoses remain rare. Table 1 summarises the demographic characteristics of the 23 participants included in the analysis.

**Table 1:** Sample characteristics (*N* = 23)

Participants	Young adults (*n* = 10)	Older adults (*n* = 13)
	*n* (%)	*n* (%)
**Sex**		
Male	6 (60)	6 (46.15)
Female	4 (40)	7 (53.85)
**Age group (years)**		
18–39	10 (100)	/
≥ 60	/	13 (100)
**Diagnostic methods for LCRCI**		
Self-perceived	10	13
Physician-confirmed	0	0

*LCRCI* long COVID-related cognitive impairment

### Characteristics of cognitive symptoms reported by participants

All participants included in the final analysis (*N* = 23) reported experiencing long COVID-associated cognitive symptoms based solely on subjective self-perception rather than formal diagnosis in hospital or primary care settings (Table 1). All participants denied any prior noticeable cognitive impairments before contracting COVID-19, attributing their symptoms solely to post-COVID-19 effects in their own perspectives, as confirmed during interviews. These cognitive symptoms did not always noticeably manifest after the initial infection; in several cases, they appeared following two or more reinfections.

**Fig. 1:**
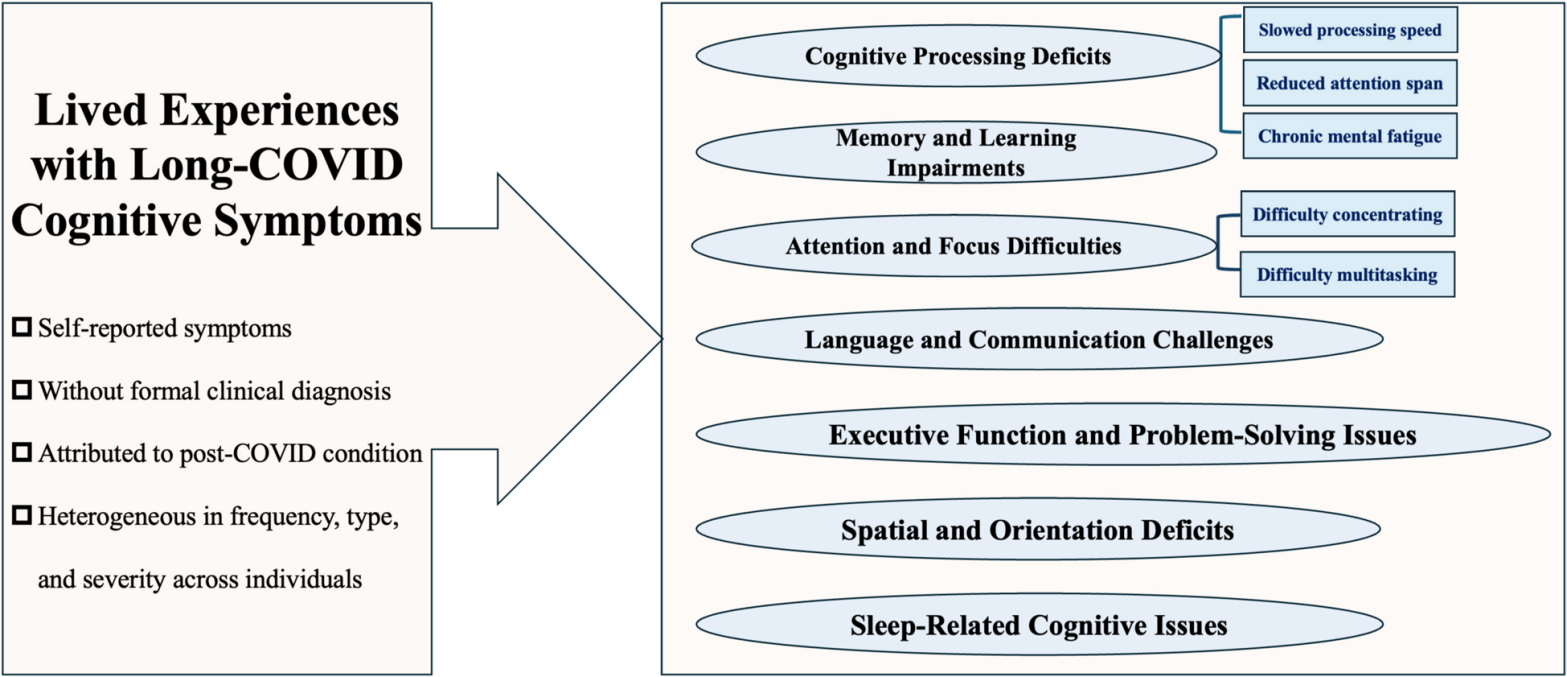
Spectrum of core cognitive symptoms reported by participants

Figure 1 illustrates the spectrum of cognitive symptoms reported by participants during interviews. These included but were not limited to memory impairment, difficulty concentrating, word-finding challenges, slowed processing speed, reduced attention span, impaired problem-solving skills, difficulty multitasking, confusion or disorientation, poor executive function (e.g. complaining about leaving food unattended as a result of losing track of time), challenges in learning new information, spatial awareness deficits (e.g. complaining about having trouble following maps, even though they were previously familiar), chronic mental fatigue. The presentation of these symptoms varied widely in frequency, type, format, and severity among individuals. Additionally, we noticed that some participants reported that sleep disturbances (e.g. insomnia or disrupted sleep patterns) could significantly trigger or worsen their cognitive issues, and that their cognition improved once their sleep quality improved. While the types of symptoms reported were broadly similar across young and older adults, with no consistent age-specific themes emerging at the group level, differences in perceived severity and frequency were visible between individuals of different ages. For transparency, an anonymised and age-stratified summary of participants’ self-reported symptoms, including perceived severity, frequency, and sleep-related issues, is provided in Additional file 1: Table S1.

In this cohort, memory impairments were among the most frequently reported symptoms in both young and older adults, primarily affecting short-term memory functions such as short-term prospective and episodic memory impairments. Participants commonly described difficulties recalling the location of personal items or remembering planned activities from recent days or hours. Sleep disturbances, particularly insomnia, were frequently cited as exacerbating these memory issues. Other commonly reported symptoms included impaired executive function in daily tasks (e.g. difficulty with meal preparation, which requires planning, organization, and multitasking), chronic mental fatigue (e.g. feeling mentally drained after routine activities like grocery shopping or attending a meeting), and verbal communication challenges (e.g. reduced verbal fluency, difficulty recalling words, and disorganised speech).

### Past management experiences with long COVID-associated cognitive symptoms

**Fig. 2:**
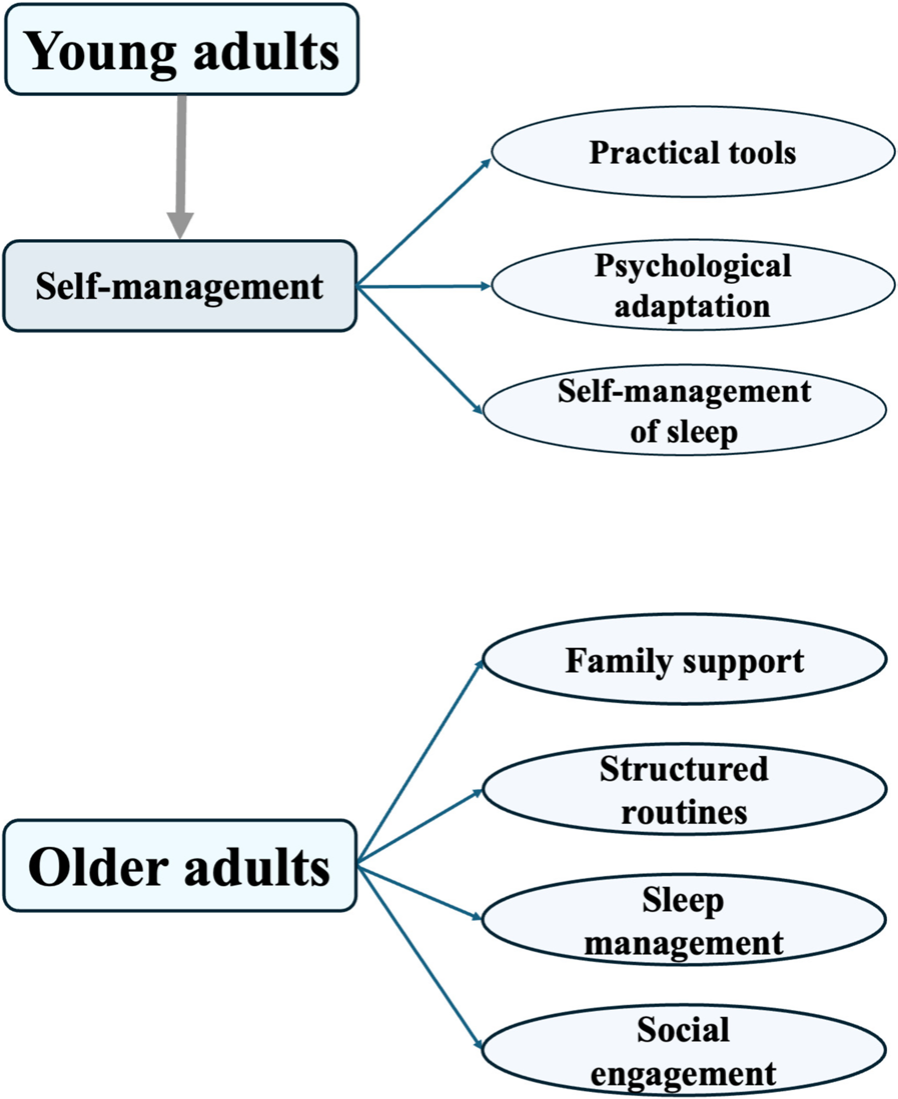
Past frequently used strategies for managing long COVID-associated cognitive symptoms by age group

The past experiences of managing long COVID-associated cognitive symptoms, including the strategies used to manage these symptoms and participants’ perceptions of their effectiveness, differed remarkably between young and older adults in this study (Fig. 2). Among young adults, over 50% of participants with symptoms did not visit a primary care provider or seek other forms of external medical care. This was often due to a subjective perception that the symptoms, though bothersome, were largely tolerable and manageable without professional intervention. Practical strategies were commonly employed, such as using tools like to-do lists or alarm clocks to aid memory. One participant described this approach: “If there’s something important, I can use a memo to write it down, or I need an alarm clock to remind me so that I don’t forget” (woman, 30 s). In addition, several participants emphasised psychological adaptation, adopting an attitude of acceptance and adjustment, particularly when the symptoms did not severely disrupt their overall functioning. Some also emphasised the important role of self-managing sleep and maintaining good sleep in supporting cognitive improvement.

For a few young adults, however, cognitive symptoms such as memory impairment were more pronounced, remarkably affecting daily life and, in certain cases, academic performance. Despite trying practical strategies, these participants reported difficulty finding effective approaches to address these challenges. One participant shared how these symptoms impacted their studies: “I’m studying [participant’s major] subject, which involves a lot of memorizations. I just can’t get that information to stick in my mind anymore. Revising has become incredibly difficult” (man, 20 s).

Older adults, on the other hand, reported distinctly different approaches to managing their symptoms. Family support played an indispensable role, with many relying on relatives to help them remember daily tasks, such as taking medication or carrying essential items. For example: “If I need to grab something later, I often forget, but my wife always reminds me” (man, 60 s). Participants also reported using environmental cues, such as placing items in visible locations, or maintaining consistent daily habits to reduce the impact of forgetfulness. For some, improving sleep was an important strategy, achieved through sleep aids or Traditional Chinese Medicine (TCM), which they believed indirectly improved cognitive symptoms. For example: “Taking medicine to regulate sleep helps. When my sleep improves, my memory is better” (woman, 60 s). Maintaining social interactions, such as brief conversations with family/community members or going for walks, was also cited as a way to stay mentally active.

### Comparison of treatment preferences and expectations between young and older adults

**Fig. 3:**
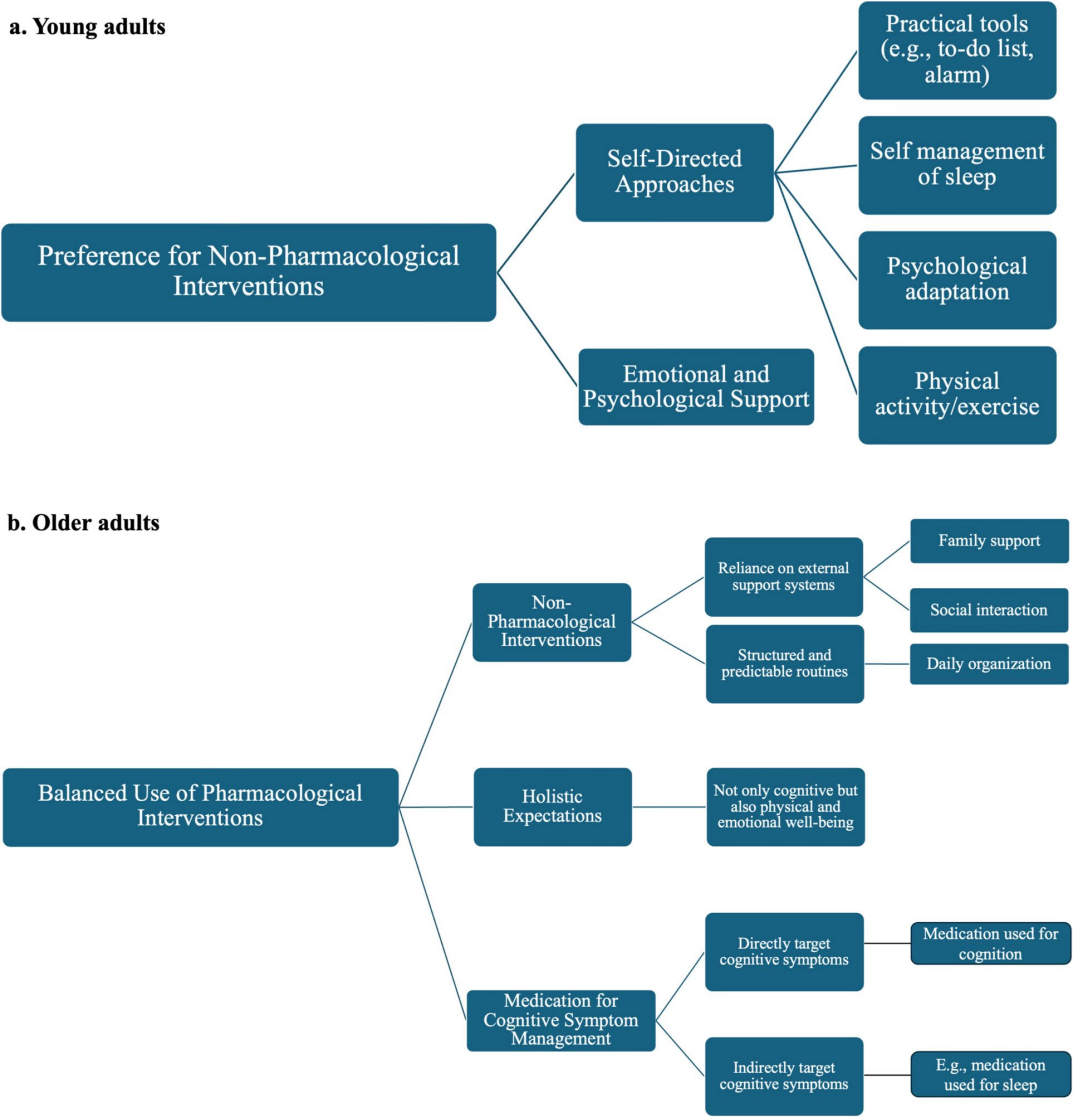
Thematic and conceptual map of age-specific preferences and expectations

The treatment preferences and expectations of young and older adults in managing long COVID-associated cognitive symptoms revealed distinct patterns. Among young adults, a central theme was a “preference for non-pharmacological interventions” (Fig. 3a), comprising two subthemes: “self-directed approaches” and “emotional and psychological support”. In contrast, the central theme for older adults was a “balanced use of pharmacological interventions” (Fig. 3b), comprising three subthemes: “non-pharmacological interventions”, “holistic expectations”, and “medication for cognitive symptom management”.

For young adults, the first subtheme, “self-directed approaches”, includes four major concepts: practical tools (e.g. to-do lists and alarms), self-management of sleep, psychological adaptation, and physical activity/exercise. Many young participants in this study expressed an expectation of minimal reliance on formal medical care, preferring self-directed strategies, particularly practical tools such as to-do lists, alarms, and digital reminders to manage memory-related issues. These tools were viewed as essential for maintaining productivity and managing daily tasks. However, exceptions were noted among those who still expected to receive formal medical care, particularly when they perceived these self-directed approaches as offering minimal or negligible benefit.

Self-management of sleep emerged as a critical concept in managing cognitive challenges among young adults, with disrupted sleep perceived both as a symptom in itself and as a factor exacerbating existing cognitive symptoms. Young adult participants described efforts to improve sleep quality through behavioural adjustments, including maintaining consistent sleep schedules, practicing relaxation techniques, and avoiding stimulants before bedtime. Psychological adaptation, such as adopting an attitude of acceptance and adjustment, was also emphasised. Physical activity, often tailored to individual capabilities, was another preferred strategy. Despite those, a few young adults did not anticipate benefits from psychological adaptation, citing its limited effectiveness as subjectively perceived, and reported a tendency to avoid physical activity or exercise. On the other hand, “emotional and psychological support” emerged as another core subtheme for young adults when dealing with cognitive symptoms. Many participants expressed a need for validation from healthcare providers and sought treatments that addressed both the cognitive and emotional impacts of their symptoms, such as stress, frustration, anxiety, and feelings of isolation.

By contrast, for older adults, three subthemes were identified, including “non-pharmacological interventions”, “holistic expectations”, and “medication for cognitive symptom management”. Reliance on external support systems, including family support and social interaction, was a critical concept in their treatment preferences and expectations. Many older participants relied on their children or spouses to remind them of daily tasks, such as taking medications or preparing meals. Maintaining social engagement, whether through brief conversations with family members or neighbourhood walks, was considered vital for both cognitive stimulation and emotional well-being. Socialization was also seen as a remedy for the isolation often accompanying long COVID symptoms. Structured and predictable routines were also favoured, as they reduced cognitive load and minimised forgetfulness. These routines, such as placing essential items in visible locations, provided a sense of order and predictability, offering comfort in the face of cognitive challenges. Moreover, older adults expressed a preference and expectation for holistic treatment approaches that addressed not only cognitive symptoms but also physical health and psychological well-being. Another critical subtheme was the use of “medication for cognitive symptom management”, which included medications targeting cognitive symptoms directly and those addressing them indirectly. Like young adults, older adults highlighted the importance of sleep in cognitive recovery. Several participants noted improvements in memory and mental clarity following treatments aimed at enhancing sleep quality, which included both prescribed medications and traditional remedies.

Overall, although the treatment preferences and expectations of young and older adults in managing long COVID-associated cognitive symptoms differed markedly, both groups highlighted the importance of improving sleep and expressed a shared desire for more holistic, individualised care that addresses both cognitive and emotional well-being.

### Theory for understanding how participants develop preferences and expectations

**Fig. 4:**
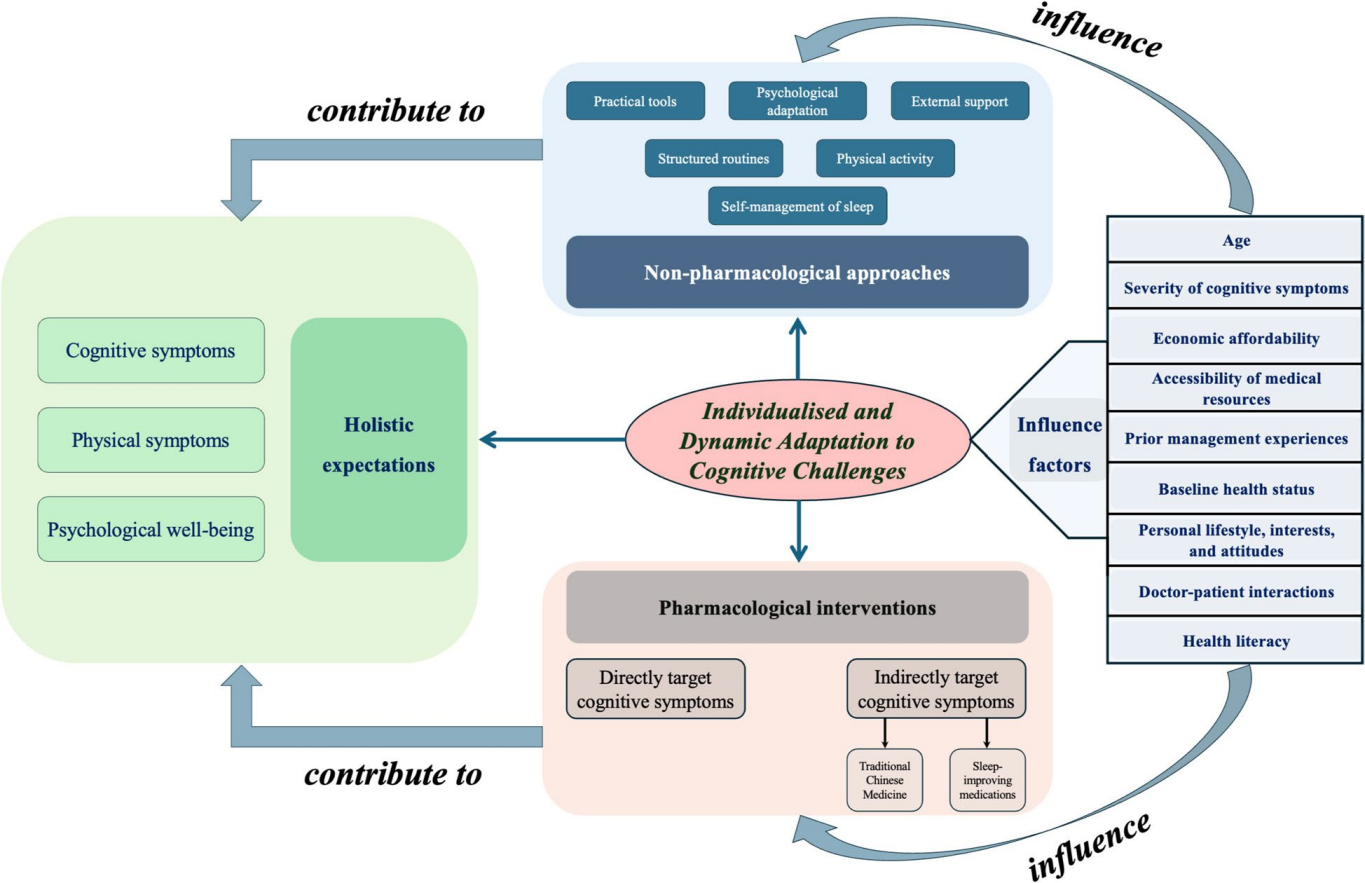
A theory of adapting to cognitive challenges in long COVID. This theoretical framework places individualised and dynamic adaptation at its centre, linking non-pharmacological approaches—such as practical tools, structured routines, physical activity, self-management of sleep, psychological adaptation, and external support—with pharmacological interventions that either directly address cognitive symptoms or indirectly support cognition (e.g. traditional Chinese medicine, sleep-enhancing medicines). A range of contextual factors—including age, severity of cognitive symptoms, economic affordability, accessibility of medical resources, prior management experiences, baseline health status, personal lifestyle, interests and attitudes, doctor–patient interactions, and health literacy—influence the selection and tailoring of these strategies. Collectively, these strategies contribute to participants’ holistic expectations across cognitive, physical, and psychological well-being, thereby shaping treatment preferences and expectations

Using a constructivist grounded theory approach, we identified a central theory: “Individualised and Dynamic Adaptation to Cognitive Challenges”, which provides insights into participants’ preferences and expectations for managing long COVID cognitive symptoms and how they are formed. As illustrated in Fig. 4, the theory reflects how individuals, both young and older adults, navigate cognitive challenges through diverse, context-driven strategies influenced by personal, social, and systemic factors.

Young adults in our study demonstrated a preference for non-pharmacological interventions to manage cognitive symptoms, while older adults tended towards a balanced approach combining pharmacological and non-pharmacological strategies. Both groups emphasised the importance of addressing psychological well-being, although older adults also expressed concerns about physical symptoms, which some attributed to age-related health decline or overall physical condition. For example, a young participant stated, “But what I feel is that the older generation, and even society as a whole, don’t seem to care much about younger people dealing with symptoms like brain fog. So, I really hope that the mental health of young people like us, who are going through long COVID, can get the attention it truly deserves” (man, 20 s). An older participant shared, “I feel like it’s not just about the brain symptoms. I really hope the physical symptoms can improve as well. After all, as we get older, our overall health tends to decline, and many symptoms often occur together. So for someone like me, as an older adult, I just hope to achieve a full recovery for my body, and of course, that includes mental health too” (man, 70 s).

Beyond age, factors such as the severity of cognitive symptoms, economic affordability, accessibility of medical resources, prior management experiences, baseline health, personal lifestyle, interests, and attitude, doctor-patient interaction, and health literacy could influence participants’ preferences and expectations.

The subjectively perceived severity of cognitive symptoms emerged as a critical factor influencing their preferences and expectations. For young adults with mild-to-moderate symptoms, self-directed strategies (e.g. psychological adaptation and practical tools) were often perceived as effective. However, those with more severe cognitive impairments found such strategies less beneficial and expressed a potential need for medical interventions, especially when supported by clear communication from trusted healthcare providers. For instance, one participant shared, “I just can’t manage it on my own. I can’t remind myself no matter what I try. I’ve tried so many things, like using memos or setting alarms, but none of it works. I still forget, no matter what. I feel like I might need some medication to make it a bit better” (woman, 30 s). Another participant highlighted the importance of doctor-patient communication, stating, “I wish the doctor could explain what to expect and give more guidance instead of just prescribing pills. Otherwise, I don’t feel very comfortable taking it, as it doesn’t seem entirely reliable to me” (woman, 60 s). Additionally, while mild-to-moderate physical activities such as walking or light jogging improved mental clarity and reduced stress for some, others avoided exercise due to concerns about post-exertional fatigue. Medications, though generally avoided, were deemed acceptable for addressing intolerable symptoms like insomnia or anxiety, which exacerbated cognitive impairments. For example, “The doctor prescribed me something to help me sleep, and after a while, I noticed I could remember things a little better” (man, 60 s).

Participants’ prior management experiences and their corresponding perceived effectiveness was another key factor. Participants were more likely to trust and adopt strategies they had found effective in the past, while tending to avoid those that were ineffective and remaining cautious about treatments with uncertain outcomes. For example, a participant explained, “If I don’t sleep well, my memory is worse the next day.So I try to go to bed and wake up at the same fixed time every day, which helps a bit. I feel like I need to stick to this routine in the future because sleep is so important to me. It is not just about memory, as it might actually improve many aspects of my life” (woman, 30 s). An older adult participant highlighted the importance of continued family support, such as from children, “I feel happy when they show care and offer their support. In the future, I hope they can visit me more often, as they did before, or take the time to call or video chat with me. Having conversations and spending time together would mean a great deal to me. As older adults, this is often what we value and hope for the most” (woman 60 s). In contrast, another participant noted their reluctance to continue ineffective treatments: “I didn’t see a doctor about my memory issues, but I tried taking trying docosahexaenoic acid (DHA) for a few months. It didn’t really help, so I stopped. And I don’t think I’ll take similar supplements in the future either, as they almost never seem to work” (woman, 30 s).

Economic affordability and accessibility of reliable medical resources also played significant roles. Several participants noted that potential financial limitations would affect their ability to pursue treatments, such as effective pharmacological options or professional therapies, when they become available. For example, one participant shared, “If there are new medications or treatment options in the future, it will depend on how expensive they are and how long the treatment will take. If it’s expensive and requires long-term treatment, it might be unaffordable. It also depends on how much the insurance can cover” (man, 70 s). Accessing reliable medical resources not only means whether the socioeconomic status of participants helps them access these resources, but also whether these resources are actually available. Many participants expressed frustration with the lack of clear guidelines and specialised clinics for managing long COVID cognitive symptoms in China. One participant remarked, “However, around here, there doesn’t seem to be any clear or formal approach to managing cognitive symptoms of long COVID. I haven’t heard of any specific places that specialize in treating this condition. It feels like no one really knows where to go for proper care” (woman, 70 s). In addition, stigma surrounding long COVID symptoms further compounded these challenges, particularly among younger adults. As one individual noted, “In real life, within my peer group, cognitive symptoms, especially those that are often subjective or less obvious, such as brain fog and mental fatigue, are frequently seen as exaggerated or even fabricated. I feel that cultural attitudes in China reinforce this bias, viewing prolonged recovery as a lack of personal effort or adaptability. This certainly adds a psychological burden to our group on top of the physical challenges of long COVID”, “People think you’re exaggerating or being lazy. It’s hard to talk about it without being judged” (man, 20 s).

Personal lifestyles choices, interests, and attitudes also influenced treatment preferences and expectations. For instance, although some participants tended to avoid exercise due to concerns about post-exertional fatigue, another potential reason could be a lack of interest in physical exercise. “I didn’t have a habit of exercising before (the COVID-19 pandemic), and I’m also worried that exercising might worsen my fatigue symptoms” (woman, 30 s). Baseline health conditions further shaped decisions, with participants managing multiple health issues tending to prioritize low-risk, non-invasive treatments. Finally, personal health literacy was also a crucial factor. We found that participants with higher health literacy, as reflected in their ability to critically assess potential treatment risks and benefits (based on our observations during interviews), were better equipped to understand and evaluate potential treatment options, further strengthening the rationale behind their treatment preferences and expectations.

## Discussion

In this study we explored the lived experiences of Chinese individuals with long COVID-associated cognitive impairments, identifying the spectrum of cognitive symptoms they reported, along with their past management experiences. We also explored their treatment preferences and expectations, with a comparative focus on young and older adults, while identifying factors beyond age that could influence these preferences and expectations. Using a constructivist grounded theory approach, we developed the central theoretical framework of “Individualised and Dynamic Adaptation to Cognitive Challenges”. This theory illustrates how factors including age, severity of cognitive symptoms, economic affordability, accessibility of medical resources, prior management experiences, baseline health, personal lifestyle, interests, and attitudes, doctor-patient interactions, and health literacy shape their preferences and expectations. Overall, our findings highlight the need for personalised, dynamic, patient-centred approaches to care.

When exploring their lived experiences, a notable finding was the reliance on self-perceived cognitive impairments rather than formal medical diagnoses among both age groups. This aligns with earlier research suggesting that cognitive symptoms such as brain fog and memory loss often go undiagnosed due to their subjective nature and the lack of standardised diagnostic criteria for long COVID-associated cognitive impairment [47]. The lack of formal diagnoses underscores the need for accessible and validated tools for assessing cognitive deficits in long COVID patients. Recent research efforts have focused on developing more objective and standardised assessment tools for the broad spectrum of long COVID symptoms, including digital screening methods and patient-reported outcome measures specifically tailored to long COVID [48, 49]. However, further validation and widespread implementation are necessary to ensure that these tools effectively support timely diagnosis and intervention, particularly for cognitive aspects. Without formal recognition, individuals could delay seeking appropriate care due to restricted access to reliable medical resources, prolonging their cognitive challenges and reducing their quality of life.

The subjective nature of self-perceived symptoms introduces variability in how individuals experience and report cognitive impairments, highlighting the need for patient-centred assessment approaches. Age-specific preferences and management strategies identified in this study underscore the heterogeneity of long COVID experiences and suggest that a one-size-fits-all approach is inadequate. Young adults predominantly relied on self-management strategies, including practical tools (e.g. to-do lists and alarms), psychological adaptation, and self-directed behavioural modifications (e.g. mild-to-moderate exercise). This preference for non-pharmacological interventions is consistent with prior research showing that younger individuals, who often possess greater cognitive resilience and physical endurance, are more likely to adopt active coping strategies [50].

However, our findings also revealed stigma associated with long COVID cognitive impairments among young adults. In China, post-COVID-19 sequelae could be perceived as poor self-adjustment or lack of resilience, exacerbating mental health burdens and further worsening cognitive symptoms, particularly for students whose academic performance is affected [51, 52]. Public health campaigns to normalize and validate the experiences of long COVID patients are necessary to mitigating these stigmatizing cultural attitudes.

In contrast, older adults prioritised structured routines and external support, with family members playing a critical role in mitigating the impact of cognitive impairments. This aligns with existing evidence showing that social and environmental support systems are vital for older adults coping with chronic conditions [53]. In our study, reliance on external support appeared closely tied to family involvement and social connectedness. While age-related physical changes may provide part of the context, participants themselves did not explicitly frame their reliance as solely stemming from physiological decline. This pattern may also be understood through frameworks such as socioemotional selectivity theory [54, 55], which emphasises older adults’ motivation to prioritise emotionally meaningful relationships and supportive interactions. At the same time, these findings may highlight the need to address caregiver dynamics in intervention strategies.

The prevalence of post-exertional malaise among individuals with long COVID presents challenges to conventional rehabilitation paradigms [56]. Although physical exercise is widely recommended for cognitive recovery in non-COVID populations, our findings suggested that such interventions may exacerbate symptoms in certain individuals. Hence, tailored treatment strategies that account for symptom profiles are essential.

Salient differences were also observed in attitudes towards pharmacological treatments between the two age groups. Younger adults expressed scepticism and a preference for non-invasive options, while older adults were more open to pharmacological interventions, provided they were part of holistic care addressing both cognitive and physical symptoms. This variation may reflect participants’ own perceptions of their broader health and medical histories, as several older adults linked their preferences to managing comorbidities and long-term medication use. Meanwhile, our findings indicated that building trust with both young and older adults is crucial for healthcare providers. Clear communication about the potential risks and benefits of pharmacological options is essential for fostering positive doctor-patient interactions, especially when patients have doubts about treatments. Furthermore, the preference for and expectation of Traditional Chinese Medicine by some older participants underscored the importance of incorporating culturally appropriate interventions into care plans, based on personal interests and attitudes.

Another notable finding was the importance of sleep management across both age groups, highlighting the potential bidirectional relationship between sleep quality and cognitive function. Poor sleep can exacerbate cognitive symptoms, creating a vicious cycle that complicates recovery. Behavioural interventions (e.g. sleep hygiene practices) and pharmacological treatments were identified as key strategies. However, younger participants often hesitated to use pharmacological sleep aids due to concerns about potential side effects. This underscores the need for non-pharmacological approaches, including cognitive behavioural therapy for insomnia (CBT-I), which has proven effective in improving sleep and reducing negative sleep-related cognitions [57–59], as well as other relaxation-based interventions (e.g. mindfulness and breathing exercises) that may be more accessible for Chinese adults.

The variability in symptom severity also influenced treatment preferences. Participants with mild-to-moderate symptoms often relied on self-management strategies, while those with severe symptoms expressed a need for professional interventions. This underscores the importance of stratified care models tailored to individual symptom severity. Economic and systemic factors, such as affordability and access to medical resources, further shaped treatment choices, particularly in settings like China, where disparities in healthcare access exacerbate the burden of long COVID [60]. Addressing these barriers is essential for equitable care.

In our study, most participants, especially older adults, were uncertain about where to seek help for long COVID-associated cognitive impairments. The role of health literacy in shaping treatment preferences and expectations suggests that educational initiatives to improve understanding of long COVID and available treatments could enhance patient engagement and adherence. Considering that older adults often have less access to reliable digital health resources and are more susceptible to misinformation compared to younger individuals [61, 62], targeted outreach programs and simplified, trustworthy health communication strategies are essential. Additionally, integrating psychological support into care plans could further address the emotional and social dimensions of long COVID-associated cognitive impairments, ultimately improving overall outcomes.

A recent systematic review found moderate-certainty evidence supporting the effectiveness of CBT and combined physical and mental rehabilitation programs for managing long COVID, with potential improvements in certain aspects of cognitive symptoms [63]. It also highlighted the potential benefits of intermittent aerobic exercise for improving physical function compared to continuous exercise, though careful tailoring is required for individuals experiencing post-exertional malaise. These findings underscore the importance of accessible and evidence-based interventions, particularly in countries like China, where public health infrastructure for long COVID is still in its nascent stages. Our study also highlighted that integrating such approaches into culturally sensitive frameworks and addressing stigma through targeted public health campaigns could enhance adoption and effectiveness. Tailored and stratified interventions are promising for optimizing outcomes across diverse patient populations.

Finally, from a clinical practice perspective, our theory of “Individualised and Dynamic Adaptation to Cognitive Challenges” highlights not only differences between age groups but also substantial variation within each group. Preferences and expectations were influenced by diverse factors such as symptom severity, prior management experiences, baseline health, health literacy, and personal lifestyle, suggesting that a one-size-fits-all approach would be inadequate. Accordingly, physicians should first discuss individuals’ specific expectations with them. For example, rather than directly recommending treatment plans that physicians believe are most helpful, physicians should first inquire about the lifestyle changes individuals may consider or prefer to change, followed by shared decision-making. Additionally, since the severity of cognitive symptoms is likely to fluctuate over time, individuals should be informed about safety-netting measures by physicians. This could include providing information on red flag symptoms to watch for and recommending periodic follow-up sessions [64]. Only through this approach can true patient-centred care be achieved.

## Strengths, limitations, and future research directions

To the best of our knowledge, this study is the first to qualitatively explore and compare age-specific treatment experiences, preferences, and expectations regarding cognitive impairments in individuals with long COVID, providing a comprehensive theoretical framework to guide the design of tailored interventions. Another key strength of this study lies in its use of the grounded theory method, employing a cyclical process of data collection and analysis through theoretical sampling. As data were analysed, emerging themes and concepts informed subsequent participant recruitment and data collection. This iterative approach ensured that underrepresented perspectives were explored and theoretical constructs refined. Interview questions were dynamically adjusted in response to evolving themes and gaps in understanding, allowing for a comprehensive theoretical framework that accurately captured the diverse and dynamic narratives of participants.

Despite its strengths, this study has several limitations. First, the reliance on self-reported cognitive symptoms without physician confirmation may introduce bias. Second, although young adults interviewed in our study reported a low prevalence of severe long COVID cognitive impairments, it is crucial to acknowledge that many individuals across the globe continue to experience intolerable cognitive symptoms [65]. This highlights the need for ongoing attention to this population. Third, although the sample was diverse in age and gender, its geographic and cultural specificity limits the transferability of the findings to broader populations. Future studies should aim to validate and complement these findings in larger, more diverse cohorts. Fourth, while theoretical sampling was a core principle of the study design, practical constraints, such as participant availability, time, and resource limitations, challenged its full implementation. To address these challenges, a pragmatic approach was adopted, integrating theoretical sampling principles within the constraints of recruitment feasibility. Fifth, we included only individuals aged 18–39 years and 60 and above, as these groups typically exhibit evident differences in baseline cognitive and physical health under normal circumstances. Future studies could include middle-aged participants to bridge this gap. Finally, the broader social context of the pandemic, including strict lockdowns and extended periods of social isolation in China, may also have contributed to participants’ perceived cognitive and psychological challenges. Previous studies have suggested that reduced social engagement and physical activity during lockdown could contribute to declines in memory, attention, and emotional well-being, particularly among older adults [66, 67]. For younger adults, restrictions on mobility and social interaction may have intensified feelings of frustration, stress, and isolation, further exacerbating cognitive difficulties [68–71]. Although participants in this study primarily attributed their symptoms to long COVID, it is possible that these contextual factors interacted with post-COVID sequelae to shape their overall experiences.

Future research should also focus on addressing key gaps in the understanding and management of long COVID-associated cognitive symptoms. First, the presence of SARS-CoV-2 in the blood is currently an imperfect indicator, as some asymptomatic individuals also exhibit signs of persistent virus [72]. This underscores the urgent need to identify reliable and effective biomarkers or measurable indicators for diagnosing long COVID cognitive impairments, especially given that some clinicians still view these symptoms as primarily psychosomatic [72]. Second, health professional training programs currently lack meaningful inclusion of long COVID-related cognitive impairments in their curricula. Prioritizing the integration of up-to-date information into training for healthcare professionals, alongside high-quality continuing education for qualified providers, is essential to improve recognition and management of these symptoms [73]. Third, quantitative research is needed to complement qualitative findings, offering broader insights into the prevalence and determinants of treatment preferences across diverse populations. Further investigation into biological mechanisms, such as neuroinflammation and vascular dysfunction, could inform the development of targeted pharmacological treatments [74]. Fourth, establishing consensus on definitions and clinical endpoints for long COVID cognitive impairments is critical to capturing their complexity and variability. Such standardised definitions will guide clinical care, research, and regulatory frameworks while advancing trials, encouraging pharmaceutical engagement, and enabling cross-study comparisons [73]. Finally, the role of caregiver support in the recovery of older adults highlights the need for interventions that address not only patient needs but also the well-being of their support networks, while also considering caregivers’ attitudes, which can significantly influence both the quality of care provided and patient outcomes.

## Conclusions

Our study highlights the multifaceted nature of managing long COVID-associated cognitive impairments, emphasizing the need for patient-centred, holistic care strategies that account for individual preferences and expectations. Tailored interventions that align with the distinct needs of both young and older adults can enhance recovery and improve quality of life for those affected. Moreover, the insights from this research provide a valuable foundation for designing future treatment trials and guiding clinical decision-making. Ensuring that interventions are not only clinically effective but also resonate with patients’ values and lifestyles will support the development of equitable, personalised care models and contribute to the effective management of this evolving public health challenge.

## Supplementary Information

Additional file 1. Table S1. Summary of participants’ self-reported cognitive symptoms, severity, frequency, and sleep-related issues.

## Data Availability

The qualitative interview data underlying this study are not publicly available because they could compromise participants’ anonymity and confidentiality. However, de-identified excerpts or coded data that support the findings are available from the corresponding author (D.S.) upon reasonable request and subject to ethical approval.

## Abbreviations

CBT: Cognitive behavioural therapy
CBT-I: Cognitive behavioural therapy for insomnia
COVID-19: Coronavirus disease 2019
DHA: Docosahexaenoic acid
MCI: Mild cognitive impairment
PLRC: Patient-led research collaborative
SARS-CoV-2: Severe Acute Respiratory Syndrome Coronavirus 2
