# Supplementary material for: Treatment experiences, preferences, and expectations for cognitive impairments in long COVID among Chinese young and older adults: a constructivist grounded theory study
Source: BMC Med. 2025 Oct 23;23:579. doi: 10.1186/s12916-025-04457-5 (PMC12548172; doi:10.1186/s12916-025-04457-5)
Supplement: Supplementary file 1 — Additional file 1. Table S1. Summary of participants’ self-reported cognitive symptoms, severity, frequency, and sleep-related issues. [file 12916_2025_4457_MOESM1_ESM.pdf]

Additional file 1 for the study 'Treatment experiences, preferences, and expectations for cognitive impairments in Long COVID among Chinese young and older adults: a constructivist grounded theory study'

Table S1. Summary of participants' self-reported cognitive symptoms, severity, frequency, and sleep-related issues (N=23)

Participant ID	Age group	Key self-reported cognitive symptoms	Perceived severity (self-description)	Symptom frequency (self-reported)	Sleep issues reported
Y01	Young adult	Short-term memory lapses, word-finding difficulty	Mild	Intermittent (several times a week)	Occasional insomnia
Y02	Young adult	Difficulty concentrating, mental fatigue	Moderate	Daily during study tasks	None
Y03	Young adult	Slowed processing speed, multitasking difficulty	Mild	Weekly fluctuations	Difficulty falling asleep
Y04	Young adult	Short-term memory loss, disorientation	Severe	Constant, every day	Frequent insomnia
Y05	Young adult	Word-finding challenges, reduced verbal fluency	Mild	Intermittent	None
Y06	Young adult	Memory impairment, attention problems	Moderate	Daily, varies with workload	Poor sleep worsens cognition
Y07	Young adult	Executive dysfunction (losing track of tasks)	Moderate	Several times a week	Disrupted sleep
Y08	Young adult	Brain fog, memory decline	Severe	Persistent, daily	Insomnia
Y09	Young adult	Forgetfulness, confusion in daily tasks	Mild	Occasional	None
Y10	Young adult	Mental fatigue, short-	Moderate	Daily	Sleep

		term memory lapses			disturbance
O01	Older adult	Memory impairment, disorientation	Moderate	Frequent, most days	Insomnia
O02	Older adult	Forgetfulness, executive dysfunction	Mild	Occasional	Uses sleep remedies
O03	Older adult	Slowed processing, attention deficit	Moderate	Daily, fluctuates	Disturbed sleep
O04	Older adult	Short-term memory loss, poor problem-solving	Severe	Constant, every day	Chronic insomnia
O05	Older adult	Attention deficit, mental fatigue	Mild	Occasional	None
O06	Older adult	Memory impairment, word-finding difficulty	Moderate	Frequent, most days	Sleep problems
O07	Older adult	Brain fog, short-term memory loss	Moderate	Daily	Sleep disturbance
O08	Older adult	Forgetfulness, confusion	Mild	Intermittent	Occasional insomnia
O09	Older adult	Executive dysfunction, mental fatigue	Severe	Persistent, every day	Insomnia worsens memory
O10	Older adult	Memory decline, multitasking difficulty	Moderate	Frequent	Sleep issues
O11	Older adult	Disorientation, short-term memory lapses	Mild	Occasional	None
O12	Older adult	Poor concentration, short-term memory loss	Moderate	Daily	Poor sleep
O13	Older adult	Forgetfulness, chronic mental fatigue	Severe	Constant, every day	Chronic insomnia

Note: This supplementary table provides an anonymised overview of participants' self-reported cognitive symptoms, self-perceived severity, frequency, and sleep-related issues. All information is based on participants' own narratives during interviews rather than standardised clinical assessments, consistent with the qualitative design of this study. "Y" refers to young adults (aged 18–39 years) and "O" refers to older adults (aged ≥60 years). The table is intended to enhance transparency and complement the thematic findings presented in the main text.
